# MicroRNA-19b Plays a Key Role in 5-Fluorouracil Resistance and Predicts Tumor Progression in Locally Advanced Rectal Cancer Patients

**DOI:** 10.3390/ijms232012447

**Published:** 2022-10-18

**Authors:** Andrea Santos, Ion Cristóbal, Jaime Rubio, Cristina Caramés, Melani Luque, Marta Sanz-Álvarez, Sandra Zazo, Juan Madoz-Gúrpide, Federico Rojo, Jesus García-Foncillas

**Affiliations:** 1 Cancer Unit for Research on Novel Therapeutic Targets, Oncohealth Institute, Health Research Institute (IIS)—Fundación Jiménez Díaz—UAM, 28040 Madrid, Spain; 2 Translational Oncology Division, Oncohealth Institute, IIS—Fundación Jiménez Díaz—UAM, 28040 Madrid, Spain; 3 Medical Oncology Department, University Hospital “Fundación Jiménez Díaz”, UAM, 28040 Madrid, Spain; 4 Pathology Department, IIS—Fundación Jiménez Díaz—UAM, 28040 Madrid, Spain

**Keywords:** miR-19b, 5-FU resistance, progression, locally advanced rectal cancer

## Abstract

The standard clinical management of locally advanced rectal cancer (LARC) patients includes neoadjuvant 5-fluorouracil (5-FU)-based chemoradiotherapy (CRT) followed by mesorectal excision. MicroRNA (miR)-19b expression levels in LARC biopsies obtained from initial colonoscopy have recently been identified as independent predictors of both patient outcome and pathological response to preoperative CRT in this disease. Moreover, it has been discovered that this miR increases its expression in 5-FU resistant colon cancer cells after 5-FU exposure. Despite the fact that these observations suggest a functional role of miR-19b modulating 5-FU response of LARC cells, this issue still remains to be clarified. Here, we show that downregulation of miR-19b enhances the antitumor effects of 5-FU treatment. Moreover, ectopic miR-19b modulation was able to restore sensitivity to 5-FU treatment using an acquired resistant model to this compound. Notably, we also evaluated the potential clinical impact of miR-19b as a predictive marker of disease progression after tumor surgery resection in LARC patients, observing that miR-19b overexpression significantly anticipates patient recurrence in our cohort (*p* = 0.002). Altogether, our findings demonstrate the functional role of miR-19b in the progressively decreasing sensitivity to 5-FU treatment and its potential usefulness as a therapeutic target to overcome 5-FU resistance, as well as its clinical impact as predictor of tumor progression and relapse.

## 1. Introduction

Colorectal cancer (CRC) remains the third most common diagnosed cancer worldwide, being one of the cancers whose incidence is increasing every year, comprising 11% of all cancer diagnoses [1,2,3]. Moreover, CRC is the second leading cause in terms of cancer mortality, accounting rectal cancer (RC) as being almost 30% of all CRC death cases [1,4]. Management of locally advanced rectal cancer (LARC) is more difficult than colon cancer due to a higher probability of spreading to other tissues, postoperative complications, and recurrence. A combined multimodal approach of fluoropyrimidine-based neoadjuvant chemoradiotherapy (nCRT) followed by total mesorectal excision (TME) represents the standard treatment for LARC patients [5,6,7,8]. Although this therapeutic approach markedly reduced the local recurrence rate from 35% to almost 5–10% [7], neither the CAO/ARO/AIO-94 nor the TME randomized phase III trials informed a benefit in overall survival rates [9,10]. These observations are substantially obtained as a consequence of several clinical advances regarding optimal treatment strategies, all aiming to optimize oncological outcomes, while minimizing associated morbidity [5]. Despite these improvements in local recurrence, a considerable number of LARC patients suffer from a poor clinical outcome due to metastatic progression, typically the liver being the first distant organ affected, followed by the lungs, even though lung metastasis is associated with shorter survival rates and appear more frequently in LARC than in colon cancer [11,12]. Therefore, the tumor progression remains as the main challenge in LARC management, with a 10-year cumulative incidence of distant metastases reported in approximately 30% to 40% of cases in recent trials [7,13,14,15,16]. In the last years, novel therapeutic approaches are based on a patient classification according to clinically defined risk categories where the MRI criteria represents a key tool for determining this issue, and include protocols with intensified pre-operative chemotherapy, such as total neoadjuvant therapy (TNT) [6,16,17]. In fact, the recently published clinical phase III trials PRODIGE 23, RAPIDO, and STELLAR have demonstrated better short- and long-term outcomes with TNT compared with standard nCRT, suggesting TNT as a novel useful clinical management option for treating LARC patients [18,19,20].

This issue highlights the urgent necessity to further understand the molecular mechanisms that govern LARC progression in order to better define the subgroup of high-risk patients that will not respond to standard nCRT. Currently, a selection of innovative nCRT protocols of LARC patients with a poor clinical outcome is currently based only on clinical parameters [13]. Thus, the implementation of robust new biological predictive markers of tumor aggressiveness and disease progression could improve the management of these cases [6,12], allowing for the accurate identification of those patients without pathological complete response (pCR) that could experiment tumor relapse. In fact, it would be of high relevance to clarify the underlying mechanisms involved in resistance to the standard 5-fluorouracil (5-FU)-based therapy used in LARC [12,21], which could be extended to CRC cases since a notable proportion of CRC patients show tumor recurrence after 5-FU treatment, mainly caused by drug-resistant cancer cells [22].

The potential clinical value of microRNAs (miRs) has progressively emerged in the last decade [21]. Several studies have reported a great variety of miRs whose deregulation is associated with cancer progression [23,24,25]. MiRs are short, single-stranded, non-coding RNAs that regulate gene expression predominantly through post-transcriptional repression, acting as tumor suppressors or proto-oncogenes depending on their target mRNAs [23,26,27]. The small size of miRs, combined with their imperfect base-pairing for target recognition, provide miRs with the capacity to play important regulatory roles in diverse pathological and physiological processes, such as cell proliferation, cell differentiation, embryonic development, apoptosis, inflammation, and tumorigenesis [27,28,29,30,31]. Over the past years, increasing evidence has demonstrated the role of altered miRs in RC progression through the regulation of certain key signaling pathways in this disease [31,32,33].

MiR-19b has been reported to be highly expressed in a wide range of hematopoietic malignancies and other solid tumor types, playing oncogenic roles through the regulation of different signaling pathways [26,34,35,36,37,38]. Interestingly, several studies have explored the clinical impact of miR-19b in CRC and LARC. Thus, miR-19b has been reported to mediate resistance to oxaliplatin treatment via SMAD4, promoting proliferation and colon cancer progression [39]. Sun and colleagues showed that exosomal miR-19b induced resistance to radiotherapy and enhanced the stemness properties of CRC cells by targeting FBXW7 [40]. MiR-19b has been proposed to play a role in 5-FU resistance of CRC cells since its expression in 5-FU resistant cells was found to be upregulated in response to 5-FU exposure in these cells [41]. Moreover, the PP2A regulatory subunit PPP2R5E has been identified as a direct target of miR-19b [26], and PP2A inhibition has been reported to determine 5-FU resistance in CRC cells, being PPP2R5E downregulation one of the contributing molecular alterations described to inhibit PP2A in this disease [42]. Molinari et al. observed that miR-17-92a-1 cluster host gene (MIR17HG) amplification, which includes miR-19b, seemed to be related to a lack of response to nCRT in LARC, suggesting the potential role that some of the miRs included in this cluster could have in response to nCRT in LARC patients [43]. In concordance, we recently reported that miR-19b deregulation is a common alteration in LARC that independently predicts both patient outcome and response to preoperative CRT [44]. Therefore, several observations suggest the potential role of miR-19b modulating 5-FU sensitivity of CRC cells, but this issue still remains to be experimentally confirmed.

In this work, we aimed to investigate the potential role of miR-19b modulating sensitivity to 5-FU treatment, as well as to further evaluate its clinical relevance in LARC, analyzing, for the first time, its potential usefulness as a predictor biomarker of disease progression. We observed that miR-19b promotes cell growth and decreases sensitivity to 5-FU treatment. Moreover, its ectopic downregulation was able to overcome 5-FU resistance using a cell line model with acquired resistance to this chemotherapy compound. Clinically, higher miR-19b levels were detected in post-neoadjuvant samples of those patients who had an absence of pathological response and, more importantly, miR-19b expression was able to anticipate patient recurrence (local or distant) in our cohort, highlighting its clinical relevance in this disease.

## 2. Results

### 2.1. miR-19b Modulates Antitumor Effects of 5-FU Treatment

**Figure 1:**
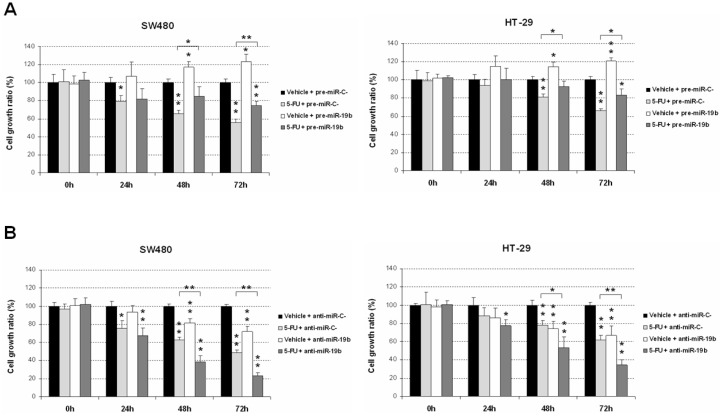
Changes in miR-19b expression results in altered sensibility to 5-FU treatment. MTS assay showing cell viability of SW480 and HT-29 cells treated with 5-FU (1 μM) and transfected with (**A**) pre-miR-19b or (**B**) anti-miR-19b; * *p* < 0.05; ** *p* < 0.01. Asterisks over the columns refer to the comparison of each condition against the control (first column).

In order to functionally confirm previous data in the literature suggesting a potential role of miR-19b regulating tumor response to 5-FU treatment, we investigate whether an ectopic modulation of miR-19b expression could alter 5-FU efficacy. As expected, we found that miR-19b overexpression significantly decreased sensitivity of both SW480 and HT-29 cells to this drug, thereby contributing to the acquisition of a more resistant phenotype. Of note, miR-19b enhanced tumor cell growth in the absence of treatment compared with normal controls in the SW480 and HT-29 cell lines (Figure 1A). To validate these findings, we next transfected both cell lines with a specific anti-miR-19b, observing an enhancement of the 5-FU-induced antitumor properties in comparison with those cells transfected with a negative control anti-miR (Figure 1B). These results would confirm that miR-19b is involved in regulating the sensitivity of CRC cells to 5-FU treatment.

### 2.2. Downregulation of miR-19b Represents a Novel Molecular Strategy to Overcome 5-FU Resistance

**Figure 2:**
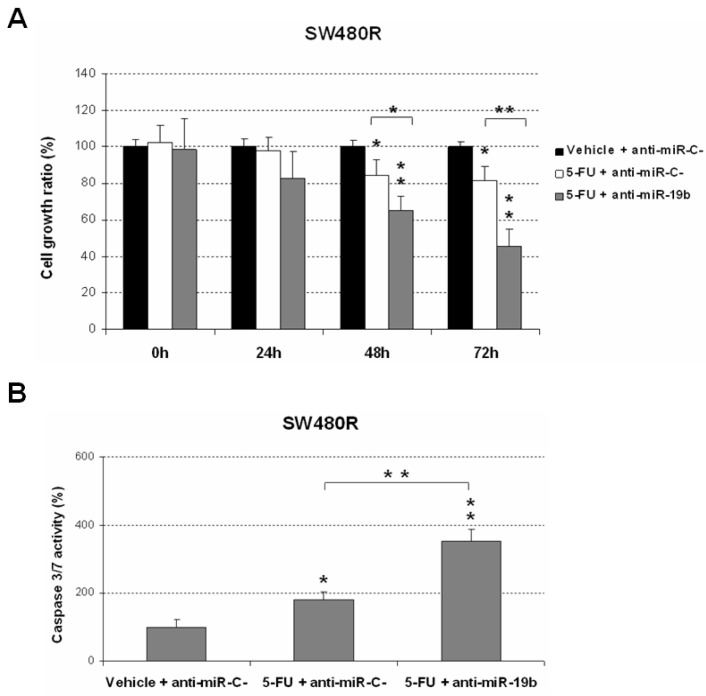
Role of miR-19b in resistance to 5-FU treatment. (**A**) MTS assay and (**B**) caspase 3/7 assay showing effects of miR-19b downregulation in SW480R cells treated with 5-FU (1 μM); * *p* < 0.05; ** *p* < 0.01. Asterisks over the columns refer to the comparison of each condition against the control (first column).

We next performed an MTS assay to explore the role of miR-19b as a novel molecular target in 5-FU resistant in LARC using and acquired resistant cell line model (SW480R). In concordance with the results described above, we observed that the ectopic downregulation of miR-19b significantly re-sensitized SW480R cells to 5-FU treatment, enhancing the antitumor effects induced by this drug (Figure 2A).

To further confirm these observations, we also performed caspase activation assays, observing that miR-19b downregulation markedly enhanced the 5-FU-induced cell apoptosis in SW480R cells (Figure 2B). 

Due to the previous data regarding the relevance of the miR-19b/PPP2R5E axis in LARC at the clinical level to determine pathological response to 5-FU-based nCRT, we aimed to experimentally confirm its role in 5-FU resistance. Notably, we observed that both ectopic PPP2R5E overexpression and the treatment with the PP2A activator perphenazine (PPZ) showed similar effects to miR-19b downregulation in overcoming 5-FU resistance. Moreover, caspase-activation assays confirmed that PPP2R5E overexpression and PPZ treatment restored 5-FU sensitivity in SW480R cells (Figure S1). Altogether, these findings would suggest that miR-19b is involved in 5-FU resistance and this miR, as well as PPP2R5E, emerges as novel targets to overcome resistance to this chemotherapy agent in LARC.

### 2.3. High miR-19b Levels Are Observed in Post-Treatment Samples from Those LARC Patients with Lack of Response to Preoperative CRT

**Figure 3:**
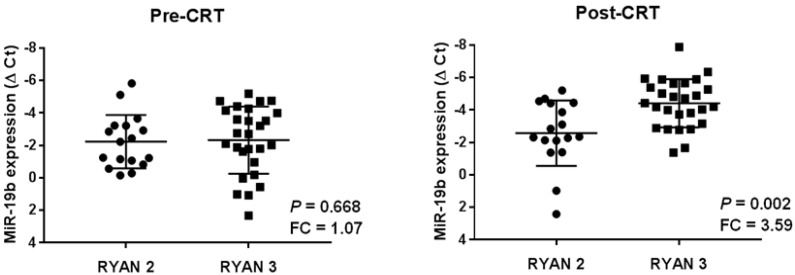
Dot-plots showing miR-19b expression in pre- and post-treatment samples from our cohort of LARC patients (*n* = 44). FC = fold change. Patients with low and high miR-19b expression levels are indicated by black squares and circles, respectively.

In order to evaluate the potential clinical impact of miR-19b in the disease progression, we quantified miR-19b expression levels in a series of 44 LARC patients with enough material available of paired pre- and post-neoadjuvant CRT samples. Clinical characteristics of the global cohort are presented in Table S1. We first stratified our cohort in those cases with minimal response with residual cancer outgrown by fibrosis (RYAN 2), or lack of response with minimal or no tumor kill and extensive residual cancer (RYAN 3). Despite the fact we observed similar miR-19b expression in pre-treatment samples between RYAN 2 and 3 subgroups (*p* = 0.668), this miR showed more than a three-fold higher expression in post-treatment samples from those cases with an absence of response to neoadjuvant CRT (*p* = 0.002) (Figure 3).

To confirm these results, we also analyzed separately RYAN 2 and 3 subgroups by scatter plots, comparing miR-19b expression levels between pre- and post-operative CRT samples from each patient. In concordance with the findings described above, we observed a significant miR-19b overexpression in post-treatment patient samples only from the RYAN 3 subgroup (Figure S2).

### 2.4. miR-19b Overexpression in Post-Neoadjuvant CRT Samples Anticipates Patient Recurrence

**Figure 4:**
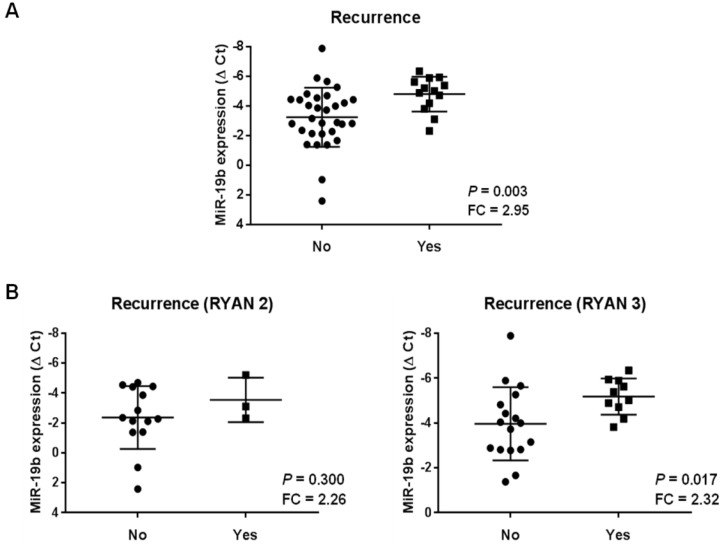
(**A**) MiR-19b expression levels in patients with and without disease relapse in the global cohort and (**B**) after stratification by RYAN status. Patients with low and high miR-19b expression levels are indicated by black squares and circles, respectively.

To further clarify the clinical significance of miR-19b expression as a novel predictor biomarker in LARC, we evaluated the status of this miR in disease progression analyzing post-CRT samples. Thus, we first studied our global cohort and observed significant higher miR-19b levels (almost a three-fold change) in those cases who had recurrence (this term includes local or distant progression) (*p* = 0.003) (Figure 4A). However, taking into account that these findings could be due to a stronger correlation between patient recurrence and RYAN 3, we next analyzed the association between miR-19b and recurrence after stratifying our series by RYAN. Notably, we detected miR-19b overexpression associated with recurrence independently of the RYAN status. Although a marked significance was reported for RYAN 3 cases (*p* = 0.017), it was not achieved in the RYAN 2 cases. It was probably due to the low number of cases who relapsed in this patient subgroup (*p* = 0.300), since both subgroups had similar fold change differences in miR-19b expression (around 2.3-fold change) (Figure 4B).

These observations prompted us to generate a ROC curve in order to establish a cutoff point for miR-19b expression in order to study its usefulness as a predictive marker of patient recurrence in post-treatment samples from LARC patients who did not respond to neoadjuvant therapy. The ROC curve obtained showed that miR-19b expression levels yielded an area under the curve (AUC) of 0.682 (95% confidence interval (CI) = 0.493 to 0.871) with 58.3% specificity and 81.3% sensitivity in discriminating LARC patient recurrence (Figure S3). Interestingly, we observed that miR-19b overexpression significantly determined patient recurrence in our cohort (*p* = 0.002), and only 13.3% of patients with low miR-19b levels relapsed (Table 1).

We next explored the association of miR-19b overexpression with local progression and distant metastasis, observing significance in both cases (*p* = 0.004 in local and *p* = 0.009 in distant recurrence) (Table S2).

Finally, we found that even though in pre-CRT samples there were no differences in miR-19b expression between RYAN 2 and RYAN 3 (Figure 3), greater expression of this miR can be detected in those cases who are going to relapse. However, in this case the association failed to achieve statistical significance (*p* = 0.095) (Figure 4).

## 3. Discussion

The current need of useful predictive biomarkers in LARC is not restricted only to those that determine the response to neoadjuvant CRT. In fact, the identification of molecular alterations involved in the disease progression that could anticipate patient recurrence would help clinicians to improve the management of LARC patients. In the last years, several miR signatures have been progressively proposed to predict pathological response in LARC, but they have not been incorporated into the clinical routine, highlighting that the identification of robust markers is still a major challenge in this disease [45,46]. In this regard, our research group recently reported that miR-19b deregulation in initial biopsies derived from colonoscopy prior to neoadjuvant therapy is a common alteration in LARC patients that associates with pathological response to preoperative CRT. Moreover, it was also described that low miR-19b levels predicted both longer overall and disease-free survival [44]. The potential clinical impact of miR-19b in LARC was hypothesized based on its regulatory function over the PP2A regulatory subunit PPP2R5E [26], whose alteration was previously described as a molecular mechanism to inhibit PP2A in CRC [42]. PP2A inhibition has been reported to be a major regulator of the sensitivity of CRC cells to standard chemotherapeutic agents, such as oxaliplatin and 5-FU [47]. This issue prompted us to propose miR-19b deregulation as a potential alteration with significance in LARC, since the standard therapy used in this disease is based on 5-FU. In addition, it has also been described in an upregulation of miR-19b in CRC drug-resistant cells in response to 5-FU treatment [41], which, indirectly, would also be contributing to our hypothesis about miR-19b in LARC. In the present work, we have demonstrated the role of miR-19b negatively regulating 5-FU sensitivity of CRC cells (Figure 1). Moreover, we validated these findings using a specific pre-miR-19b that resulted in decreased 5-FU-derived antitumor effects, further confirming the role of this miR in modulating sensitivity to this drug. Notably, we showed that this miR represents a plausible molecular target to overcome resistance to this drug in LARC patients with a lack of response to standard preoperative CRT, since an ectopic miR-19b downregulation was able to restore 5-FU sensitivity using an acquired resistant model to this compound (Figure 2). Our observations would suggest that this miR could also have both a clinical and therapeutic impact in metastatic CRC (mCRC), since 5-FU is widely used within the adjuvant therapy in the clinical management of these patients. Moreover, miR-19b has been reported to mediate oxaliplatin resistance through a direct regulation of SMAD4 and NR3C1 [39,48]. In concordance with these findings, it has also been described in the role of exosomal miR-19b secretion modulating oxaliplatin sensitivity in CRC cells [49]. Altogether, these observations, together with the fact that 5-FU plus oxaliplatin regimens have been used in recent Phase III TNT trials, such as PRODIGE 23, RAPIDO, and STELLAR [18,19,20], reinforces the potential relevant role of this miR in determining the efficacy of this therapeutic approach in LARC and also in mCRC patients, but this issue needs to be experimentally clarified.

At the clinical level, it has been reported that miR-19b associates with metastasis and a poor outcome in metastatic CRC patients [28], suggesting its potential role in mediating disease progression in LARC. Therefore, we examined paired pre- and post-neoadjuvant CRT samples from a cohort of LARC cases who did not respond to preoperative treatment and, therefore, tumor material could be obtained in the surgical resection. These cases were stratified considering a minimal (RYAN 2) or a total lack of response and tumor progression (RYAN 3). This study aimed to evaluate whether miR-19b could serve as a marker of disease progression in post-surgery specimens, in addition to its previously reported value in initial biopsies as a predictor of pathological response to neoadjuvant CRT. Thus, we found differential expression only in post-treatment samples, with miR-19b significantly overexpressed in RYAN 3 cases compared to the RYAN 2 subgroup (Figure 3). We also detected higher levels of this miR in those cases that experienced disease relapse. Furthermore, after stratifying our patient cohort by RYAN, the analysis excluded that this association could be due to the higher miR-19b expression levels in RYAN 3 cases (Figure 4). Therefore, we generated a ROC curve and demonstrated the clinical value of the obtained cutoff to predict patient recurrence in our LARC cohort (Table 1). However, the low number of patients included in our cohort, as well as the lack of cases with RYAN 0 and 1 and its retrospective character, represent important limitations of this study that have to be considered, and conclusions must be considered with caution until a proper validation in a larger independent series be carried out. Furthermore, it remains necessary to fully investigate the functional role of the targets regulated by miR-19b with special interest in PPP2R5E regarding LARC, since previous data demonstrated its inverse correlation in initial biopsies from LARC patients together with its predictive value of response to preoperative CRT [44], and the results showed in the present work confirm its relevance in the 5-FU resistance phenotype (Figure S1). Of interest, PPP2R5E downregulation was previously described as a molecular contributing alteration to inhibit PP2A in CRC [42]. Importantly, the activation status of the PP2A pathway plays a key role in regulating 5-FU sensitivity in this disease, which would explain the effects observed after PPP2R5E modulation and also with the treatment with a PP2A activator, such as PPZ. Another issue that will also have to be evaluated is the potential usefulness of miR-19b as circulating marker in LARC liquid biopsies. In fact, a recently published study has identified this miR within a group of six miRs with an upregulated expression after CRC resection [50], which would indicate its clinical value in this disease and LARC. All of these considerations will need to be clinically validated before the inclusion of miR-19b in clinical protocols.

## 4. Materials and Methods

### 4.1. Cell Cultures and Transfection

The human CRC cell lines SW480 (ATCC CCL-228) and HT-29 (ATCC HTB-38) were purchased from the American Type Culture Collection (ATCC, Manassas, VA, USA). Authentication was carried out by the authors in all cases (LGC Standards, Wesel, Germany). The medium used was RPMI-1640 (Invitrogen, Carlsbad, CA, USA) supplemented with 10% fetal bovine serum (FBS), penicillin G (100 U/mL), and streptomycin (0.1 mg/mL), and cell lines were grown at 37 °C in a 5% CO_2_ atmosphere. Cells were treated with 5-FU (Calbiochem, San Diego, CA, USA) at the indicated concentrations for each experimental condition. For transfection experiments, CRC cells were seeded in 6-well plates and transfected with 10 µL of Lipofectamine 2000 (Invitrogen, Carlsbad, CA, USA) and 20 nM of a miR-19b specific mirVana^TM^ miRNA Mimic and Inhibitor (Ambion, Cambridge, UK). The 5-FU resistant cells were generated by culturing SW480 cells in the presence of increasing doses of the drug (three subculturing doses per concentration), starting at 0.1 µM. In order to assess the evolution of resistance, we determined IC50 after every 5-FU concentration point, by using an MTS assay (Promega, Madison, WI, USA) after 24 h of treatment. The resistance of every 5-FU-resistant clone was defined as the ratio between resistant and parental cells IC50 values.

### 4.2. Patient Samples

All of the samples used in this research study were kindly supplied by Fundación Jiménez Díaz biobank (Madrid, Spain). A cohort of 44 consecutive specimens from patients with a histological diagnosis of LARC who were treated with preoperative CRT between 2007 and 2017 at the University Hospital Fundación Jiménez Díaz, (Madrid, Spain) were retrospectively selected for this work. We included in this work all of the samples from our previous work [44], which contained enough post-neoadjuvant CRT material in order to evaluate disease progression parameters. All cases had an accurate preoperative locoregional staging based on magnetic resonance image (MRI) of the pelvis and/or transrectal ultrasound (TRUS). A full body computed tomography scan (FBCTS) was carried out in all patients in order to exclude metastatic disease. The patients were treated with chemoradiotherapy regimens based on 5-FU, and underwent surgery 6 to 8 weeks after neoadjuvant CRT completion. All participants gave written informed consent for tissue storage and analysis at Fundación Jiménez Díaz biobank (Madrid, Spain). The ethical committee institutional review board of Fundación Jiménez Díaz University Hospital reviewed and approved the project (ref. 2018/54).

The tumor specimens obtained from the initial biopsies by colonoscopy and those derived from surgical resection were classified according to the College of American Pathologist guidelines for invasive carcinomas (TNM, 7th ed.). Two independent pathologists who were blinded to patient outcome evaluated tumor regression grade according to the modified Ryan classification, as previously described [44]. According to the clinical guidelines, every regression grade was compared with the primary tumor [51].

### 4.3. Nucleic Acid Isolation

Isolation of total RNA from formalin-fixed paraffin-embedded (FFPE) tumor samples was performed using the RecoverAll Total Nucleic Acid Isolation kit (Thermo Fisher Scientific, Waltham, MA, USA) according to the manufacturer’s instructions. The FFPE tumor sections were obtained from the interior of the paraffin block in order to avoid potential contaminations or nucleic acid damage during their storage. For the RNA isolation procedure, we used 2 consecutive 10 µm sections from tumor blocks of each patient cut in a microtome. The sections were included in positive charged glass slides, and tumor areas were carefully selected by a pathologist. We next removed the non-tumor circundant tissue prior to starting the nucleic acid isolation. The total RNA obtained was quantified by measuring its absorbance at 260 nm in a NanoDrop Spectrophotometer (Thermo scientific, Waltham, MA, USA).

### 4.4. Quantification of MiRNA Expression Levels

Samples were reverse transcribed using the TaqManHMicroRNA Reverse Transcription Kit (Applied Biosystems, Foster City, CA, USA) and mature miRs were quantified by quantitative real-time reverse transcription polymerase chain reaction (RT-PCR) using TaqMan MicroRNA Assays (Applied Biosystems, Foster City, CA, USA) specific for miR-19b (reference number: 000396) and U6B (reference number: 001093) as an internal control. Reactions were carried out using an Applied Biosystems 7500 Sequence Detection System. Conditions: 95 °C for 10 min, followed by 45 cycles of 95 °C for 15 s, and 60 °C for 1 min. The analysis of relative gene expression data was performed using the ΔCT method [52].

### 4.5. Cell Viability Assay

Cell proliferation was measured in triplicate wells by the MTS assay in 96-well plates using the CellTiter 96 Aqueous One Solution Cell Proliferation Assay (Promega Corp, Madison, WI, USA), according to the manufacturer’s instructions.

### 4.6. Analysis of Caspase Activation

Caspase-3/7 activities were quantified using the caspase Glo-3/7 assay kit (Promega Corp, Madison, WI, USA), following the manufacturer´s recommendations. The intensity of the luminescent signal was measured by a FLUOstar OPTIMA luminometer (BMG Labtech, Cary, NY, USA). Differences in caspase-3/7 activity are expressed as fold change in luminescence.

### 4.7. Statistical Analysis

The SPSS20 for Windows (SPSS Inc., Chicago, IL, USA) and GraphPad Prism v7.0 (GraphPad Software Inc., San Diego, CA, USA) bioinformatics tools were used to perform the statistical analyses of this study. We applied the χ^2^ test (Fisher exact test) based on bimodal distribution of data to analyze correlations between miR-19b and the clinical and pathological variables. Comparisons of miR-19b expression between tumor samples from each patient before and after preoperative CRT were carried out using Mann–Whitney and paired t tests. This work was carried out in accordance with Reporting Recommendations for Tumor Marker Prognostic Studies (REMARK) guidelines [53]. Data represented for transfection experiments are the mean of three independent experiments ± s.d. Statistical comparisons were carried out by 2-sided *t*-test analyses. A *p*-value less than 0.05 was considered statistically significant.

## 5. Conclusions

In summary, miR-19b plays a functional role in regulating the sensitivity of CRC cells to 5-FU treatment. Moreover, ectopic miR-19b downregulation emerges as a novel therapeutic strategy to overcome the 5-FU resistant phenotype. In addition, we observed that miR-19b deregulation shows a clinical impact in LARC patients and could be a useful predictor marker in disease progression, anticipating those cases that will develop tumor recurrence. Altogether, our findings highlight the potential relevance of miR-19b as a novel marker and molecular target in LARC, which has to be validated in forthcoming works, including additional experimental confirmation, as well as analysis in larger independent patient cohorts.

## Figures and Tables

**Table 1:** Association between patient recurrence and miR-19b expression in post-treatment biopsies from LARC patients.

Recurrence	No. Cases	No (%)	Yes (%)	*p*
miR-19b expression	44	32	12	0.002
No	30	26 (86.7)	4 (13.3)	
Yes	14	6 (42.9)	8 (57.1)	

## Data Availability

Data sharing is not applicable for this article.

## Supplementary Materials

The following are available online at https://www.mdpi.com/article/10.3390/ijms232012447/s1.
