# Supplementary material for: MicroRNA-19b Plays a Key Role in 5-Fluorouracil Resistance and Predicts Tumor Progression in Locally Advanced Rectal Cancer Patients
Source: Int J Mol Sci. 2022 Oct 18;23(20):12447. doi: 10.3390/ijms232012447 (PMC9604503; doi:10.3390/ijms232012447)
Supplement: Supplementary file 1 [file ijms-23-12447-s001.zip › ijms-1947072-supplementary.pdf]

MicroRNA-19b plays a key role in 5-fluorouracil resistance and predicts tumor progression in locally advanced rectal cancer patients

Andrea Santos [1, 2,§], Ion Cristóbal [1,2,§,*], Jaime Rubio [1,3], Cristina Caramés [1,3], Melani Luque [4], Marta Sanz-Alvarez [4], Sandra Zazo [4], Juan Madoz-Gúrpide [4], Federico Rojo [4] and Jesús García-Foncillas [2,3,*]

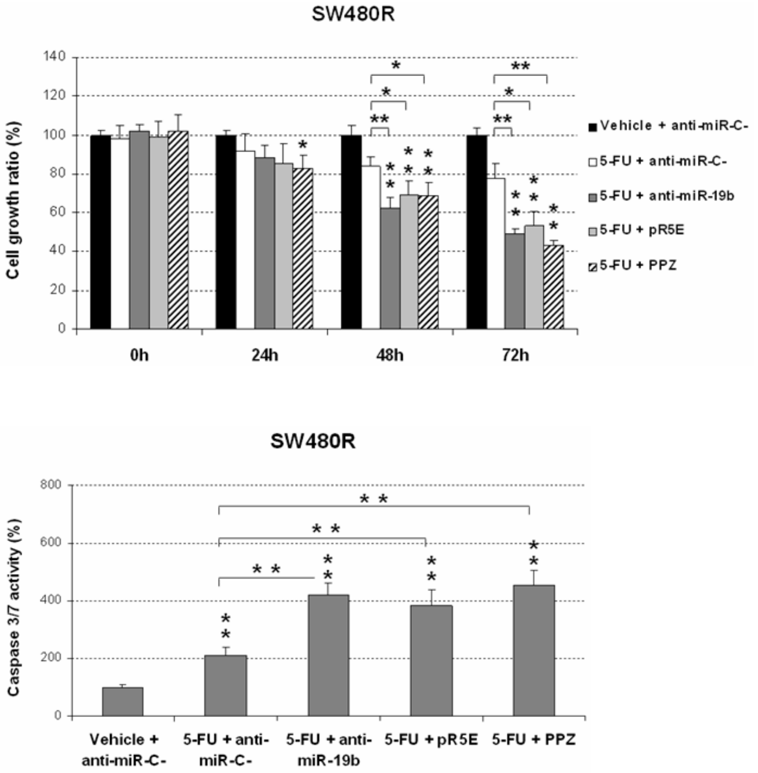

Figure S1. MTS and caspase-activation assays showing that the miR-19b/PPP2R5E signaling axis emerges as a novel theraprutic target to overcome 5-FU resistance; *P < 0.05; **P < 0.01. Asterisks over the columns refer comparison of each condition against control (first column).

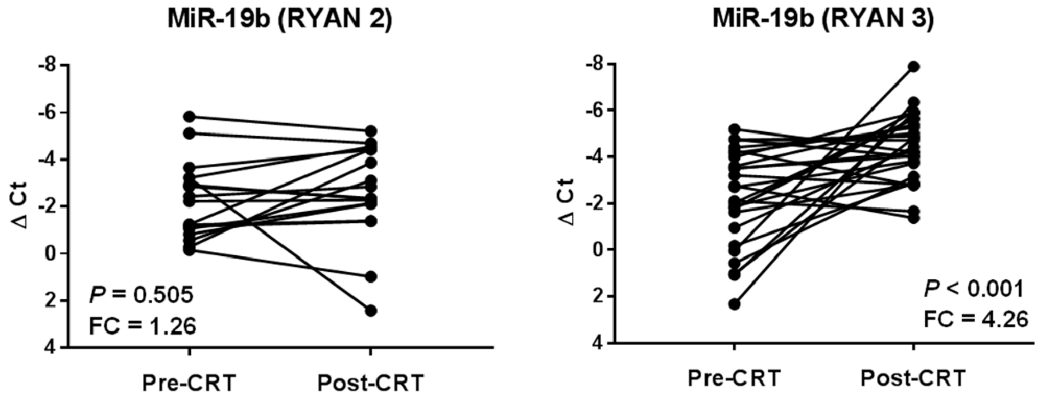

Figure S2. Scatter plots showing miR-19b expression in pre- and post-preoperative CRT samples from each LARC patient of our global cohort. FC = fold change.

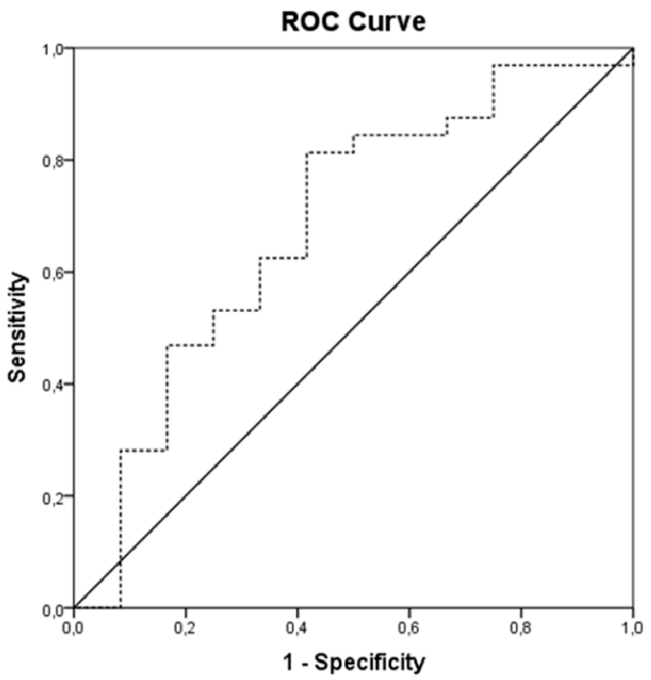

Figure S3. Receiver operating characteristic (ROC) curve to assess the usefulness of miR-19b to discriminate patient recurrence in post-treatment samples from LARC patients that have a lack of response to neoadjuvant CRT. The dashed line represents the coordinated points of the ROC curve. The solid line is the diagonal reference line.

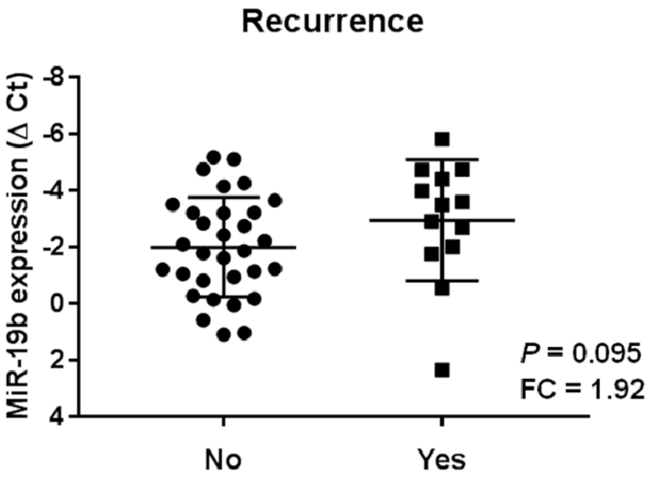

Figure S4. MiR-19b expression levels in pre-CRT samples from LARC patients with and without disease relapse in our global cohort. FC = fold change.

Table S1. Clinical characteristics of a series of 44 LARC patients with paired pre- and post-neoadjuvant CRT biopsies available.

		No.	(%)
Sex			
	Male	28	(63.6)
	Female	16	(36.4)
Age			
	<70	27	(61.4)
	≥70	17	(38.6)
ECOG[1]			
	0	29	(65.9)
	≥1	15	(34.1)
Site of primary tumor			
	Rectum	44	(100)
Clinical stage pre-CRT[2]			
	II	3	(6.8)
	III	41	(93.2)
Grade pre-CRT			
	Low	18	(40.9)
	Moderate-High	26	(59.1)
Pathological response			
	RYAN 2	17	(38.6)
	RYAN 3	27	(61.4)

[1]ECOG = Eastern Cooperative Oncology Group; [2]CRT = Chemoradiotherapy; RYAN 2 = residual cancer outgrown by fibrosis; RYAN 3 = minimal or no tumor kill and extensive residual cancer.

Table S2. Association between local and distant recurrence and miR-19b expression in post-treatment biopsies from non-responder LARC patients to neoadjuvant CRT.

**Parameter**	**No. Cases**	**No**	**(%)**	**Yes**	**(%)**	***p***
MiR-19b overexpression	44	30		14		
Local recurrence	44	30		14		**0.004**
No	38	29	(76.3)	9	(23.7)	
Yes	6	1	(16.7)	5	(83.3)	
Distant metastasis	44	30		14		**0.009**
No	33	26	(78.8)	7	(21.2)	
Yes	11	4	(36.4)	7	(63.6)	
